# Spinal Anesthesia for Cesarean Delivery: Does Ropivacaine Offer Hemodynamic Advantages Over Bupivacaine?

**DOI:** 10.7759/cureus.84809

**Published:** 2025-05-25

**Authors:** Gunjan Wadhwa, Umesh Kumar Singh, Rishi Anand, Bhanu Pratap Swain, Deb Sanjay Nag, Biswajit Sen

**Affiliations:** 1 Anesthesiology, Tata Main Hospital, Jamshedpur, IND; 2 Anesthesiology, Manipal Tata Medical College, Jamshedpur, IND

**Keywords:** bupivacaine, cesarean section, motor block, ropivacaine, sensory block, spinal anesthesia

## Abstract

Background: The selection of a local anesthetic for spinal anesthesia (SA) during cesarean sections (CSs) is a crucial clinical decision, with bupivacaine being the most frequently used agent. Nonetheless, ropivacaine has emerged as a viable alternative because of its advantageous safety profile. This study sought to compare the efficacy and safety of hyperbaric ropivacaine (0.75%) and hyperbaric bupivacaine (0.5%) in elective CS.

Methods: A prospective, randomized, double-blind, controlled trial was conducted involving 98 American Society of Anesthesiologists (ASA) II parturients who underwent elective CS. Participants were assigned to receive either 2.6 mL of 0.75% hyperbaric ropivacaine (Group R, n = 49) or 0.5% hyperbaric bupivacaine (Group B, n = 49). The primary outcome measured was the duration of motor blockade, assessed using the modified Bromage scale. Secondary outcomes included the onset of sensory block, hemodynamic stability, adverse effects, and neonatal appearance, pulse, grimace, activity, and respiration *(*APGAR) scores.

Results: Both study groups displayed similar demographic and surgical profiles. Bupivacaine exhibited a more rapid sensory onset at T10 (p = 0.004) and extended the duration until the first request for analgesia (p < 0.001). Conversely, ropivacaine was associated with significantly fewer hemodynamic fluctuations, notably a reduction in hypotension (p = 0.007). The duration of motor blockade and neonatal outcomes were comparable between the groups.

Conclusion: Bupivacaine demonstrated a marginally faster onset of sensory effects and prolonged duration of analgesia, whereas ropivacaine exhibited superior hemodynamic stability, characterized by a reduced incidence of hypotensive episodes. Both anesthetic agents were equally effective in achieving motor blockade and providing optimal surgical conditions. These findings suggest that ropivacaine is a viable alternative to SA for CSs, particularly in patients with hemodynamic vulnerabilities.

## Introduction

The prevalence of cesarean sections (CSs) has been progressively rising across various regions and countries, posing a significant challenge to healthcare providers. According to the most recent data, approximately 21.1% of all births worldwide are conducted via CS, a substantial increase from approximately 7% in 1990 [1]. Considering this increase, optimizing surgical procedures, including anesthesia selection, is of paramount importance. While general anesthesia (GA) remains an option for CSs, spinal anesthesia (SA) offers distinct advantages for both the mother and fetus due to the reduced risk of complications [2]. Several hospital-based studies have identified SA as the preferred anesthesia technique among parturients [3,4]. SA typically involves intrathecal administration of a local anesthetic agent. The most commonly used commercially available local anesthetic drugs for SA are bupivacaine and ropivacaine. The hyperbaric preparation of local anesthetic drugs, which are denser than cerebrospinal fluid, is preferred in SA because of the consistent post-spinal sensory and motor blockade outcomes [5]. Although bupivacaine (1-butyl-N-(2,6-dimethylphenyl) piperidine carboxamide hydrochloride) remains the most used hyperbaric local anesthetic, the recent commercial availability of hyperbaric ropivacaine (2S)-N-(2,6-dimethylphenyl)-1-propylpiperidine-2-carboxamide) has generated considerable interest within the anesthesiology community [6,7]. Ropivacaine has gained recognition as a safe and promising local anesthetic agent for SA due to its potential for hemodynamic stability and its ability to facilitate faster regression of blockade, thereby enabling early mobility.

A literature review indicated that intrathecal ropivacaine is associated with a shorter duration of sensory block and a reduced degree of motor block compared to intrathecal bupivacaine [8]. Furthermore, intrathecal ropivacaine reportedly elicits fewer hemodynamic side effects than bupivacaine [9]. Although both bupivacaine and ropivacaine have been used for SA in CSs, direct comparisons of their hyperbaric formulations are limited in the literature [10]. This gap in the literature highlights the need for further research to evaluate the efficacy and safety of these anesthetic agents in this clinical context. To address this gap, the current study aimed to provide a direct comparison between hyperbaric ropivacaine and hyperbaric bupivacaine in elective CSs, focusing on their efficacy and safety profiles.

## Materials and methods

Research question

Is there a difference in the onset and duration of sensory and motor block when using 0.75% Hyperbaric ropivacaine compared with 0.5% hyperbaric bupivacaine in SA for adult women (age ≥ 18 years) undergoing elective CS? 

Aim of study

This study aimed to compare the efficacy of 0.75% hyperbaric ropivacaine with 0.5% hyperbaric bupivacaine in SA for cesarean delivery, specifically focusing on the onset and duration of sensory and motor block.

Study design

This prospective, randomized, double-blind, controlled trial compared the efficacy of 0.75% hyperbaric ropivacaine and 0.5% hyperbaric bupivacaine for SA in 98 women undergoing elective cesarean delivery. This study was conducted at the Tata Main Hospital, Jamshedpur, India, from July 2023 to June 2024. Approval was obtained from the Institutional Ethics Committee of the Tata Main Hospital prior to the study. The clinical trial was registered under the registration number IRCT 20221109056455N1. Written informed consent was obtained from all participants prior to enrollment.

Participants

**Figure 1:**
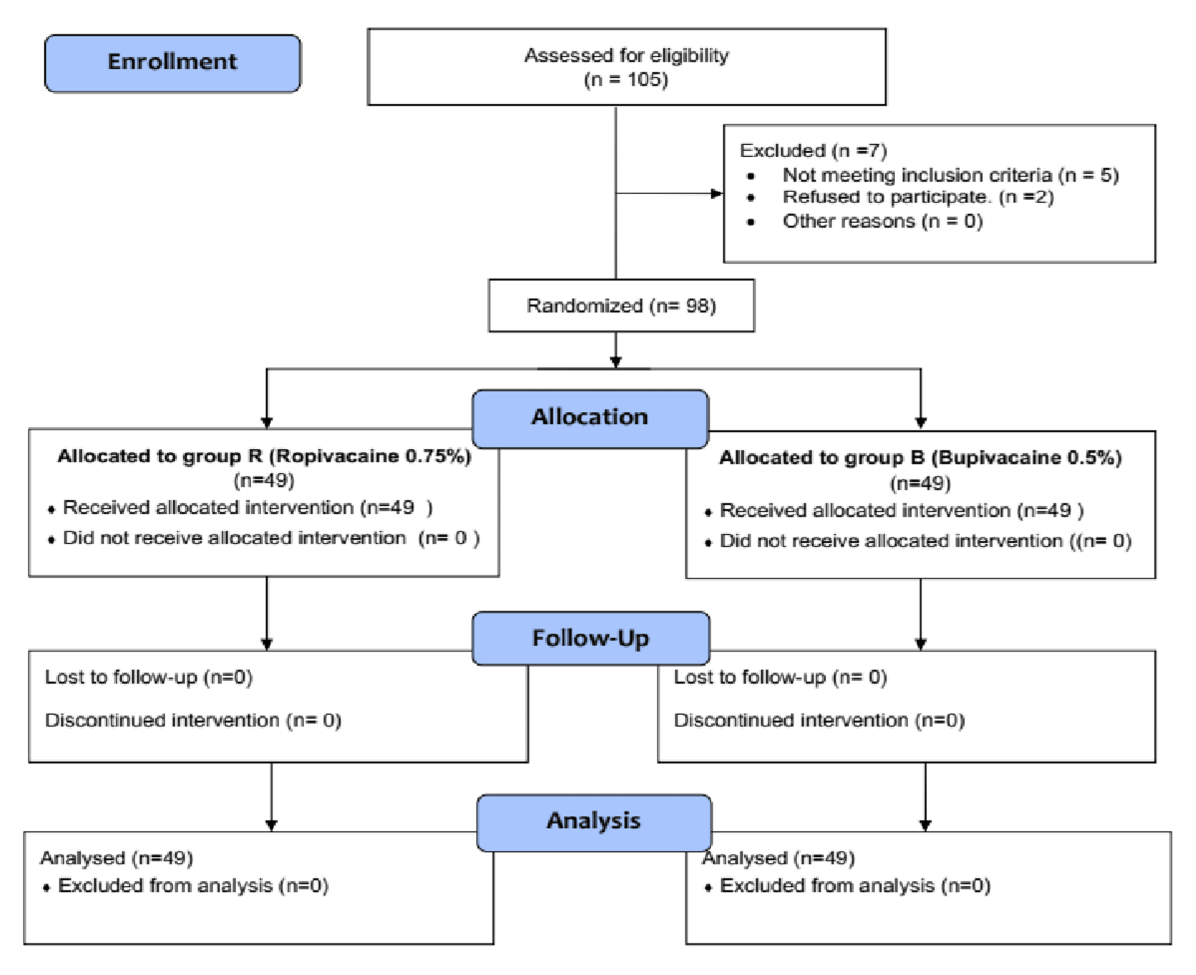
CONSORT diagram: participant flow through the trial CONSORT: Consolidated Standards of Reporting Trials

The study population consisted of adult pregnant women (≥18 years) scheduled for elective cesarean delivery under SA and classified as ASA physical status II. The exclusion criteria encompassed preterm pregnancy (<37 weeks of gestation), obstetric complications (including psychiatric, neurologic, cardiac, or hematologic disease, diabetes, multiple gestation, preeclampsia, eclampsia, bleeding, or coagulation disorders), evidence of fetal compromise, height <150 cm or >180 cm, body mass index (BMI) ≥ 30 kg/m², and a history of allergy to ropivacaine or bupivacaine. A total of 98 participants were randomly assigned to two groups for this study, as illustrated in the participant flow diagram (Figure 1).

Procedures

Participants were randomly assigned to one of two groups using a sealed envelope method, with envelopes prepared by an independent researcher not involved in the study execution. An anesthesiologist not affiliated with the study administered the spinal block, ensuring that participants remained unaware of their treatment allocation. A designated pharmacist ensured that the study drug ampules were uniformly labeled.

Participants in the ropivacaine group (Group R) and bupivacaine group (Group B) were administered 2.6 ml of 0.75% hyperbaric ropivacaine and 2.6 ml of 0.5% hyperbaric bupivacaine, respectively. The dosages of ropivacaine and bupivacaine were determined based on their demonstrated efficacy in prior studies and institutional protocols, with the objective of achieving optimal analgesia while minimizing adverse effects [11].

SA was performed in the sitting position using a midline approach, typically at the L3-4 interspace, with a 27-gauge Quincke spinal needle. The local anesthetic was injected at approximately 0.2 ml/second. Participants who required positions other than supine were excluded. Standard intraoperative monitoring, including electrocardiography, noninvasive blood pressure measurement, and pulse oximetry, was performed.

Outcome measures

The primary outcome was the duration of motor blockade, which was evaluated using the modified Bromage scale (0-3) [12]. Secondary outcomes encompassed the onset of sensory block to the T6 dermatome (assessed via cold touch), the onset of motor block (using the modified Bromage scale), the quality of abdominal muscle relaxation (categorized as 4, excellent; 3, satisfactory; 2, unsatisfactory; 1, poor) as determined by the surgeon, the incidence of intraoperative pain or discomfort (managed with intravenous fentanyl 50 mcg boluses as required), the occurrence of intraoperative complications (including hypotension, bradycardia, chest pain, dyspnea, nausea, and vomiting), neonatal appearance, pulse, grimace, activity, and respiration (APGAR) scores at one minute and five minutes, the duration of sensory block regression to the L5 dermatome (assessed via cold touch), the time to the first request for postoperative analgesia, and the incidence of postoperative complications (such as headache, back pain, and transient neurological symptoms).

The level of sensory blockade was evaluated using a swab soaked in cold water at 30-second intervals for the initial 10 minutes, followed by assessments at 15 and 20 minutes postspinal injection, and subsequently at 30-minute intervals until regression to the L5 dermatome was observed. Motor blockade was assessed using the modified Bromage scale (0, no blockade; 3, complete blockade) at 30-second intervals for the first 10 minutes, then at 15 and 20 minutes, and subsequently every 30 minutes until full recovery was achieved. Hypotension, defined as a mean arterial pressure decrease greater than 25% from baseline or a systolic blood pressure below 100 mmHg, was managed with intravenous boluses of 6 mg of mephentermine. Bradycardia, characterized by a heart rate below 60 bpm, was treated with 0.6 mg intravenous atropine only if accompanied by hypotension or chest pain. Surgeon-rated abdominal relaxation was assessed using a four-point scale (1, poor; 2, average; 3, good; 4, excellent) at the end of the surgery.

Statistical analysis

Data analysis was conducted using Jamovi statistical software (The Jamovi Project, 2025, Version 2.6). Continuous variables were evaluated for normality using the Shapiro-Wilk test. For continuous variables exhibiting normal distribution, comparisons were made using the independent samples t-test, whereas non-normally distributed variables were analyzed using the Mann-Whitney U test. Categorical variables were compared using the chi-square test. Statistical significance was set at p < 0.05. Prior to the study, a formal sample size calculation was performed to ascertain the requisite number of participants based on the primary outcome measure (duration of motor blockade) and data from preceding studies. According to previous data [13], 90 participants (45 per group) were required to detect a difference between the two groups, with an effect size of d = 0.77, power of 95%, and significance level of 5% (two-tailed). To account for potential dropouts, we enrolled 98 participants. G* Power software was used for sample size determination [14].

## Results

Patient demographics

**Figure 2:**
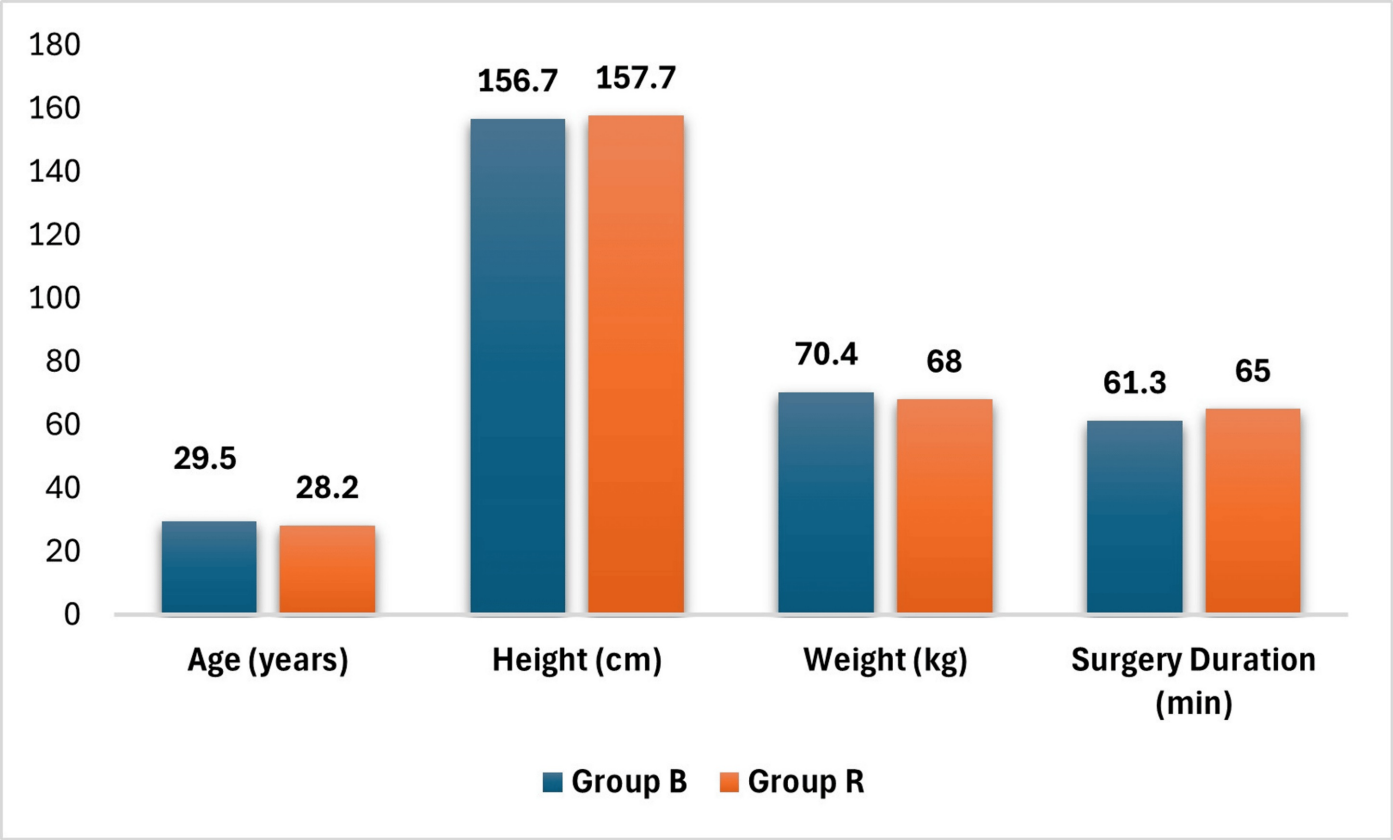
Patient demographics and surgery duration Y axis: data expressed as mean values. X axis: age (years), height (centimeters), weight (kilograms), surgery duration (minutes). Group B: bupivacaine 0.5% hyperbaric group; Group R: ropivacaine 0.75% hyperbaric group

The study involved 98 patients evenly distributed into two groups of 49, with similar demographic characteristics (age, height, and weight) and surgical durations (all p > 0.05; Table 1, Figure 2).

**Table 1:** Baseline demographic and clinical characteristics Data are presented as mean ± standard deviation (SD); independent sample t-test was used. Group B: bupivacaine 0.5% hyperbaric group; Group R: ropivacaine 0.75% hyperbaric group

Characteristic	Group B (N = 49)	Group R (N = 49)	Test statistic (t)	p-value
Drug administered	Bupivacaine 0.5%, 2.6 ml hyperbaric	Ropivacaine 0.75%, 2.6 ml hyperbaric		
Age, years	29.5 ± 3.8	28.2 ± 4.0	1.737	0.086
Height, cm	156.7 ± 7.2	157.7 ± 7.6	-0.721	0.473
Weight, kg	70.4 ± 10.9	68.0 ± 11.0	1.087	0.28
Surgery duration, min	61.3 ± 12.8	65.0 ± 15.4	-1.290	0.2

Hemodynamic parameters

**Figure 3:**
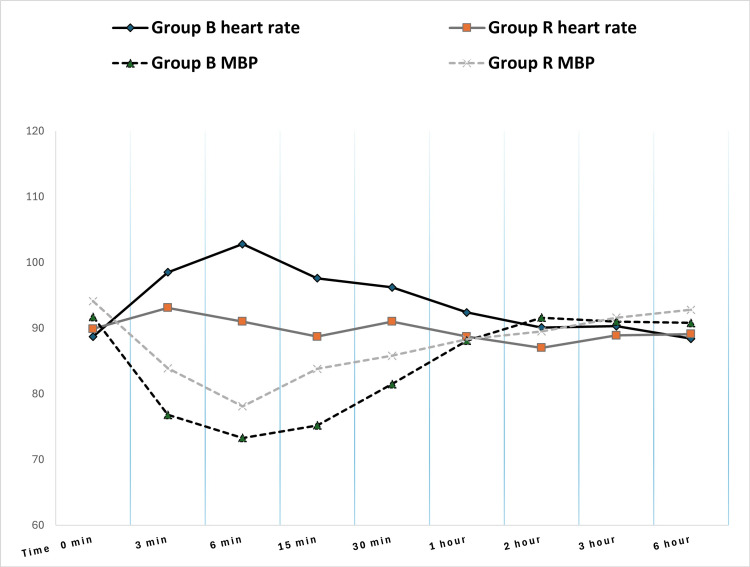
Patient hemodynamics: heart rate and mean blood pressure X axis: indicates time interval. Y axis: heart rate (HR) in beats per minutes, mean blood pressure (MBP) in mm of Hg. Group B: bupivacaine 0.5% hyperbaric group; Group R: ropivacaine 0.75% hyperbaric group

Hemodynamic assessments revealed transient yet significant differences: the bupivacaine group demonstrated elevated heart rates at six minutes (t = 3.037, p = 0.003) and 15 minutes (t = 2.349, p = 0.021), as well as reduced mean blood pressure (MBP) at three minutes (t = -3.375, p = 0.001), six minutes, 15 minutes (t = -5.286, p < 0.001), and 30 minutes compared to the ropivacaine group (Table 2, Figure 3). These hemodynamic findings underscore the clinical relevance of choosing an appropriate anesthetic agent.

**Table 2:** Intraoperative and postoperative hemodynamic parameters over time Data are presented as mean ± standard deviation (SD). An independent sample t-test was used. Group B: bupivacaine 0.5% hyperbaric group; Group R: ropivacaine 0.75% hyperbaric group

	Heart rate (beats per minute)				Mean blood pressure (mm Hg)			
Timepoint	Group B	Group R	Test statistic (t)	p-value	Group B	Group R	Test statistic (t)	p-value
	(N = 49)	(N = 49)			(N = 49)	(N = 49)		
0 min	88.7 ± 12.5	89.9 ± 11.6	-0.521	0.604	91.7 ± 9.6	94.1 ± 8.2	-1.327	0.188
3 min	98.5 ± 13.7	93.1 ± 13.9	1.925	0.057	76.8 ± 9.2	83.9 ± 11.6	-3.375	0.001
6 min	102.8 ± 19.4	91.0 ± 18.9	3.037	0.003	73.3 ± 7.9	78.1 ± 11.5	-2.392	0.019
15 min	97.6 ± 17.3	88.7 ± 20.1	2.349	0.021	75.2 ± 7.9	83.8 ± 8.3	-5.286	<0.001
30 min	96.2 ± 16.2	91.0 ± 14.9	1.655	0.101	81.5 ± 7.7	85.8 ± 9.3	-2.517	0.013
1 hour	92.4 ± 10.9	88.7 ± 10.7	1.708	0.091	88.1 ± 7.7	88.3 ± 9.2	-0.144	0.886
2 hours	90.1 ± 10.9	87.0 ± 8.2	1.578	0.118	91.6 ± 8.6	89.5 ± 9.5	1.187	0.238
3 hours	90.3 ± 9.4	88.9 ± 7.5	0.844	0.401	91.0 ± 7.5	91.6 ± 9.5	-0.319	0.75
6 hours	88.4 ± 10.0	89.1 ± 7.5	-0.411	0.682	90.8 ± 8.6	92.8 ± 7.5	-1.274	0.206

Block characteristics

Bupivacaine exhibited a more rapid sensory onset at T10 (U = 808, p = 0.004) and a prolonged duration until the first analgesia request (U = 527, p < 0.001), although motor block characteristics were comparable between the groups (Table 3). The duration of sensory and motor blockade appeared to be shorter with ropivacaine; however, this difference was not statistically significant (p > .05).

**Table 3:** Block characteristics and analgesia duration MB1: Modified Bromage scale 1; MB2: Modified Bromage scale 2; MB3: Modified Bromage scale 3. Data are presented as mean ± standard deviation (SD). Mann-Whitney U tests were used for non-normally distributed data. Group B: bupivacaine 0.5% hyperbaric group; Group R: ropivacaine 0.75% hyperbaric group

Parameter	Group B (N = 49)	Group R (N = 49)	Test statistic (U)	p-value
Sensory onset (T10), sec	51.4 ± 34.1	86.9 ± 62.8	808	0.004
Sensory onset (T6), sec	167.1 ± 71.2	195.9 ± 91.7	1018	0.188
Motor blockade onset (MB1), sec	53.9 ± 38.7	63.7 ± 43.6	1042	0.247
Motor blockade onset (MB2), sec	102.4 ± 64.2	118.8 ± 72.7	1011	0.169
Motor blockade onset (MB3), sec	167.3 ± 65.9	202.0 ± 103.0	991	0.134
Time to first analgesia (min)	240.6 ± 80.0	173.0 ± 58.4	527	<0.001
Sensory regression to L5, min	389.2 ± 77.3	379.4 ± 102.3	1109	0.513
Motor blockade regression to MB1, min	229.2 ± 77.8	215.0 ± 73.2	1063	0.324

Adverse events were more frequent with bupivacaine, particularly hypotension (χ² = 7.34, p = 0.007), whereas the incidence of bradycardia and nausea/vomiting did not differ significantly between the two groups (Table 4). The surgeons reported excellent muscle relaxation in all cases across both groups. Neonatal APGAR scores were satisfactory in both groups.

**Table 4:** Adverse events χ² tests for categorical data; values are expressed as numbers of events n (%). Group B: bupivacaine 0.5% hyperbaric group; Group R: ropivacaine 0.75% hyperbaric group

Event	Group B (N = 49)	Group R (N = 49)	Test statistic (χ²)	p-value
Hypotension	37 (75.5%)	24 (49.0%)	7.34	0.007
Bradycardia	7 (14.3%)	13 (26.5%)	2.26	0.133
Nausea/vomiting	10 (20.4%)	14 (28.6%)	0.883	0.347

## Discussion

SA is a prevalent choice for CSs because of its efficacy and safety profile. It is extensively utilized in both elective and emergency cesarean deliveries, with varying prevalence rates across different regions and healthcare settings [15,16]. Hyperbaric bupivacaine is frequently employed because of its effective anesthetic properties and capacity to provide reliable SA with a controlled spread of the anesthetic block [17]. The comparison between ropivacaine and bupivacaine for SA has been studied, focusing on their efficacy and safety in non-obstetrical and obstetrical settings using different concentrations, baricities, and adjuvants [9,10]. Both anesthetics are effective but differ in their block characteristics, side effects, and patient recovery profiles. Considering the recent launch of commercial preparations of ropivacaine in India, this study undertook a comparative analysis of hyperbaric ropivacaine (0.75%) and hyperbaric bupivacaine (0.5%) for SA in elective CSs, focusing on block characteristics and hemodynamic effects. The two groups were well-matched in terms of baseline demographics and surgical duration, ensuring comparability.

The comparative efficacy and safety of bupivacaine and ropivacaine in spinal anesthesia have been extensively studied, with our current findings contributing new insights to the existing literature [9,10,18]. When examining hemodynamic stability, sensory/motor block characteristics, and clinical outcomes, our results both confirmed and contrasted with previous meta-analyses in important ways.

Hemodynamic stability

Our study demonstrated significantly higher hypotension rates with bupivacaine (75.5%) than with ropivacaine (49.0%, p = 0.007), a finding more pronounced than that in Jaafarpour et al.'s (relative risk (RR), 1.57, p = 0.3) or Anand et al.'s (RR, 0.86; p = 0.58) meta-analyses [10,18]. This stronger association may reflect our specific surgical population, standardized dosing protocol, and higher dosage used. Similar to Khalil et al., we observed a greater hemodynamic impact of bupivacaine; however, our hypotension incidence was substantially higher than their reported standardized mean difference (SMD) of 0.43, possibly due to differences in patient population subsets or fluid management protocols [9].

Sensory and motor block characteristics

Our sensory block findings align with those of Anand et al., showing faster T10 onset with bupivacaine (p = 0.004). However, while Anand reported significantly faster motor recovery with ropivacaine (SMD -1.24), our study found more comparable motor block durations, similar to Jaafarpour's obstetric data [18]. We observed that ropivacaine showed a slower onset and faster recovery, but the difference was small and not significant. The absence of significant differences in the duration of motor blockade may be ascribed to the comparable baricity, heightened sensitivity of the obstetrical population to local anesthetics, and the 0.75% concentration of ropivacaine employed in the study [19].

Adverse events

Previous meta-analyses have reported a lower incidence of nausea and vomiting, though statistical significance was not achieved (RR, 0.85; p = 0.86), as well as shivering (RR, 0.81; p = 0.58) [10]. We observed numerically lower rates with ropivacaine (20% vs. 30%), although these differences did not reach significance. This trend reflects the hemodynamic advantage observed in previous studies. Our bradycardia rates (10% vs. 15%) showed less divergence than those in Khalil's analysis at higher doses (p = 0.09), possibly due to our fixed dosing regimen [9]. Neonatal outcomes were consistent with those of Jaafarpour et al.; we found no significant differences in APGAR scores at one minute and five minutes, reinforcing the obstetric safety of both agents [18]. Our surgical condition ratings, which were excellent in 95% of cases in both groups, suggest broad applicability and acceptance.

Clinical implications

The data presented herein reinforce existing recommendations while introducing additional nuance. The hemodynamic advantage of ropivacaine appears more pronounced in our obstetrical population compared to other obstetric studies, indicating a particular benefit for pregnant patients. Although bupivacaine's superiority in sensory block is consistent across various settings, the higher incidence of hypotension observed in our study suggests caution in short-duration procedures, day care surgical setups, and obstetrical procedures. The similar adverse event profiles of both agents support their use when neonatal outcomes are the primary concern. These findings underscore the influence of patient population, surgical context, and specific protocols on the trade-offs between bupivacaine and ropivacaine identified in previous meta-analyses.

Limitations

The single-center design of the study may limit the generalizability of its findings. Weight-adjusted dosing considers individual patient characteristics, enabling more precise and personalized treatment. This approach may result in better efficacy and fewer side effects compared to fixed dosing regimens. Further research is necessary to assess the impact of weight-adjusted dosing on clinical outcomes across various patient populations and therapeutic areas. Additionally, extended postoperative follow-up periods may be required to identify variations in recovery profiles and long-term outcomes in mothers and neonates.

## Conclusions

This study demonstrates that 0.75% hyperbaric ropivacaine serves as a clinically effective alternative to 0.5% hyperbaric bupivacaine for SA in elective CSs. Although bupivacaine is associated with a more rapid sensory onset and extended analgesia, ropivacaine offers superior hemodynamic stability, evidenced by a significantly reduced incidence of hypotension, rendering it preferable for patients with elevated cardiovascular risk. These distinctions highlight the influence of drug selection, dosing, and study design on clinical outcomes. For optimal patient care, anesthetic choice should be individualized; ropivacaine is recommended for hemodynamically vulnerable parturients, while bupivacaine is preferable when prolonged postoperative analgesia is a priority. Future research should focus on investigating standardized dosing regimens to further enhance efficacy while minimizing adverse effects. These findings, in conjunction with existing evidence, underscore the importance of tailored anesthesia strategies based on patient-specific factors and surgical requirements.
